# The Nighttime Fragrance of *Guettarda scabra* (Rubiaceae): Flower Scent and Its Implications for Moth Pollination

**DOI:** 10.3390/molecules28176312

**Published:** 2023-08-29

**Authors:** María Cleopatra Pimienta, Diego Salazar, Suzanne Koptur

**Affiliations:** 1 Department of Biological Sciences, International Center for Tropical Botany, Institute of the Environment, Florida International University, Miami, FL 33199, USA; mpimi007@fiu.edu; 2 Department of Biological Sciences, Binghamton University, Binghamton, NY 13902, USA; dsalazaramor@binghamton.edu

**Keywords:** flower scent, GC–MS, night-blooming plant, pine rockland, scent localization, volatile organic compounds, VOCs

## Abstract

Floral scent is crucial for attracting pollinators, especially in plants that bloom at night. However, chemical profiles of flowers from nocturnal plants with varied floral morphs are poorly documented, limiting our understanding of their pollination ecology. We investigated the floral scent in *Guettarda scabra* (L.) Vent. (Rubiaceae), a night-blooming species with short- and long-styled floral morphs, found in the threatened pine rocklands in south Florida, US. By using dynamic headspace sampling and GC–MS analysis, we characterized the chemical profiles of the floral scent in both morphs. Neutral red staining was also employed to determine the specific floral regions responsible for scent emission in *G. scabra*. The results revealed that *G. scabra*’s fragrance consists entirely of benzenoid and terpenoid compounds, with benzeneacetaldehyde and (*E*)-β-ocimene as dominant components. There were no differences in the chemical profiles between the long- and short-styled flowers. Staining assays indicated that the corolla lobes, anthers, and stigma were the primary sources of the scent. These findings indicate that *G. scabra*’s floral scent is consistent with that of night-blooming plants pollinated by nocturnal hawkmoths, providing important insights into its chemical ecology and pollinator attraction. This study demonstrates how floral scent chemistry can validate predictions based on flower morphology in hawkmoth-pollinated plants.

## 1. Introduction

Floral scent is one of the key traits that plants use to achieve successful reproduction, because it contributes to attracting pollen vectors and thus promotes cross-pollination [1,2]. The emission of floral scent is particularly important for night-blooming plants that depend on flower visitors for pollen transfer [3]. At night, visual cues become less effective over long distances, making chemical cues essential in attracting pollinators close enough that both senses can be used to determine the exact location of a flower [3,4].

Scent emission usually happens right before anthesis and signals the availability of floral rewards to nearby pollen vectors [5]. Fragrance release usually peaks when the flower is receptive to pollination [6], and frequently correlates with the peak activity of moths in night-blooming hawkmoth-pollinated plants [7,8,9]. In general, floral fragrances are mixtures of volatile organic compounds (VOCs) produced by floral tissues, particularly petals [10]. These mixtures can contain a diverse array of chemical groups such as aliphatics, benzenoids, and terpenes, as well as nitrogen- and sulphur-containing compounds [2,10,11]. Most flowers produce complex odor blends that can typically contain 20–60 chemical compounds [2,12,13]. The richness and diversity of these compounds vary widely interspecifically [13] and can even differ between floral morphs within a species [14].

Flowers of different animal-pollinated plant species are characterized by specific ratios of volatile compounds, often resulting in a unique fragrance that can attract certain guilds of pollinators or even particular pollinator species [2,15]. Nocturnal flowers that are pollinated by sphingid moths have a distinctive and strong scent that is usually dominated by terpenes and benzenoids and may include small nitrogen-containing compounds [15,16,17]. In addition to the chemical composition, and along with other sensory information, the spatial distribution and concentration of floral scent results in the selective attraction of pollinators to a flower [1]. This selective targeting is reinforced by the fact that most insect pollinators learn to associate a particular floral bouquet with a specific food reward [2,10,18,19] thanks to their remarkable cognitive flexibility [20,21,22], and to link the maximum emission of particular floral VOCs to the availability of high-quality rewards [10,23]. These associations enhance flower fidelity in many insects, thereby increasing their efficiency as pollen vectors [10,23]. Such mutualistic relationships have promoted coevolutionary dynamics across plants and insects, resulting in plants targeting highly effective pollinators by including compounds that act as strong attractants to that group in their floral scent [15].

The floral scents for night-blooming plants with dimorphic flowers are poorly documented, limiting our understanding of their pollination ecology. A clear understanding of pollination ecology is particularly important in order to design and implement conservation strategies for native plants whose habitats are disjunct due to natural landscape features or severely reduced in area due to human actions. This is the case of the rough-leaf velvetseed, *Guettarda scabra* (L.) Vent. (Rubiaceae), a night-blooming species that is native to south Florida’s threatened pine rockland and hardwood hammock habitats in the US [24]. These habitats have lost most of their original coverage over the last century and are considered imperiled due to severe anthropogenic fragmentation as development has proceeded in Florida [25,26,27,28,29].

**Figure 1:**
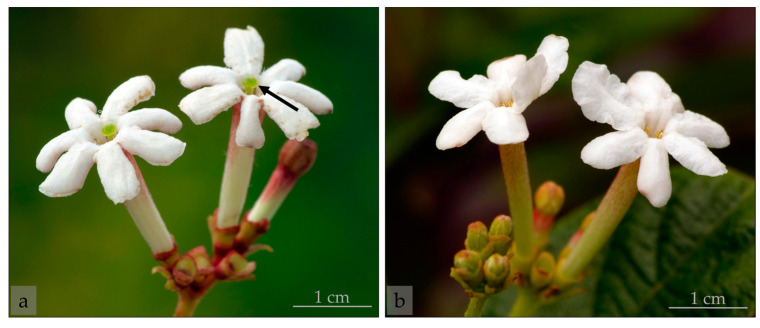
Freshly-opened flowers of *Guettarda scabra*. (**a**) The stigma in long-styled flowers is clearly visible (black arrow points to a stigma) due to the length of the style supporting it, (**b**) a characteristic not visible in short-styled morphs. Note the naturally occurring reddish color on the outside of the distal portion of the corolla tube.

*Guettarda scabra* is a night-blooming plant that is morphologically distylous, with every plant bearing either short- or long-styled floral morphs (Figure 1), a trait that may promote outcrossing via differential pollen placement on the visitor, subsequently found to be the case in many species [2]. Flowers in this species are characterized by white color, long tubular corollas (Figure 1), nectar secretion, and the emission of a strong fragrance at night. This combination of traits has long suggested that *G. scabra* is pollinated by nocturnal moths, particularly hawkmoths (Sphingidae) [2,30], an association frequently observed in species in the Rubiaceae family [31,32]. In accordance with these predictions, the pollination relationship between *G. scabra* and nocturnal hawkmoth pollinators was recently confirmed [33], as well as the flowers serving as a resource for a wide variety of arthropods [34].

Despite being an important source of floral rewards for the local arthropod fauna [34], many basic aspects of the floral ecology of *G. scabra* remain unknown, such as those related to floral scent emission and composition. To address these gaps, we set out to determine the chemical profile of the floral scent in *G. scabra* and evaluate whether this profile is conserved across floral morphs. Additionally, we aimed to determine the location of floral scent release within the flowers. Given the evidence of hawkmoth pollination in this plant, we sought to determine whether the chemical composition of *G. scabra*’s floral scent is consistent with the typical fragrance profile of a hawkmoth-attracting plant. To the best of our knowledge, this is the first reported analysis of floral volatiles in *G. scabra*, providing a baseline for understanding the chemical ecology in this species.

## 2. Results

### 2.1. Scent-Emitting Regions within the Flower

**Figure 2:**
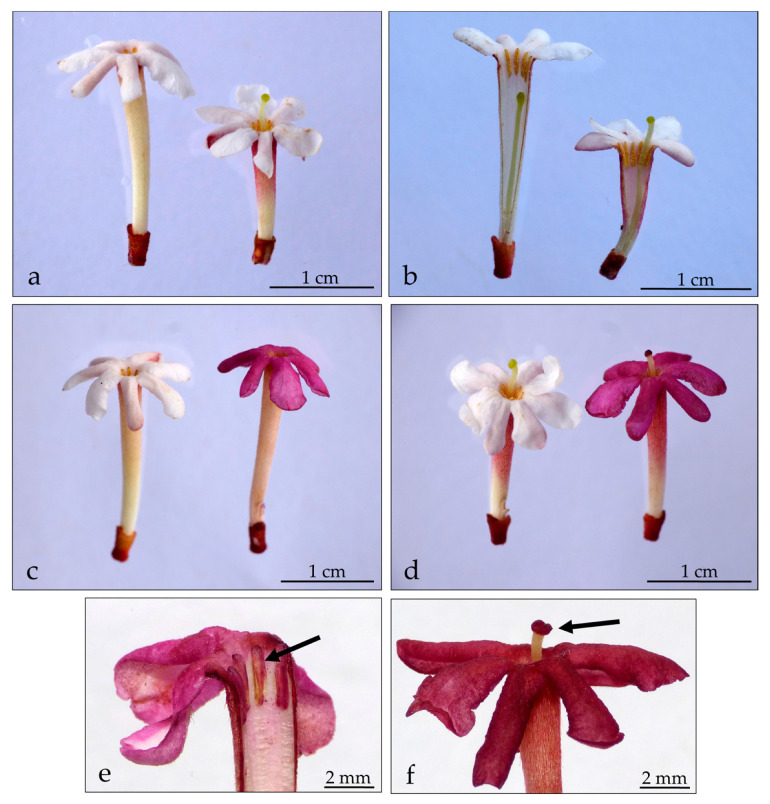
Scent-emitting areas in *Guettarda scabra* floral morphs as evidenced by treatment with neutral red. (**a**,**b**) Unstained short- and long-styled morphs. (**c**) Short-styled flowers before and after staining. (**d**) Long-styled flowers before and after staining. (**e**,**f**) Close-up showing the intense purple coloration in anthers and stigma, respectively (black arrows) after having reacted positively with neutral red. The reddish tinge on the outside of the corolla tubes in (**a**,**d**) are not stained, but rather the natural coloring of the pubescence on the outside of some floral tubes.

Neutral red staining showed that the upper surface of the corolla lobes, anthers, and stigma in both long- and short-style morphs reacted positively to the neutral red solution, suggesting that flowers of *G. scabra* might emit scent from these parts (Figure 2). The corolla tube, both inside and outside, did not react to the staining and conserved its original color.

### 2.2. Composition of Floral Scent

**Figure 3:**
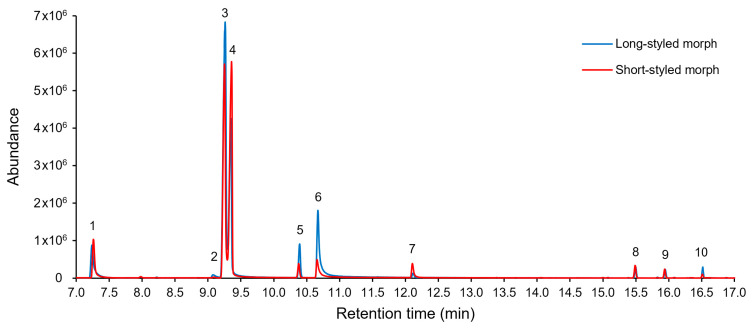
Gas chromatography–mass spectrometry (GC–MS) chromatograms of the volatile organic compounds (VOCs) emitted by long- and short-styled flowers of *Guettarda scabra*. Samples from flowers collected at ENP. Numbered peaks are identified as follows: (1) benzaldehyde, (2) benzyl alcohol, (3) benzeneacetaldehyde, (4) (*E*)-β-ocimene, (5) linalool, (6) phenylethyl alcohol, (7) methyl salicylate, (8) β-caryophyllene, (9) α-humulene, (10) (*E*,*E*)-α-farnesene.

**Figure 4:**
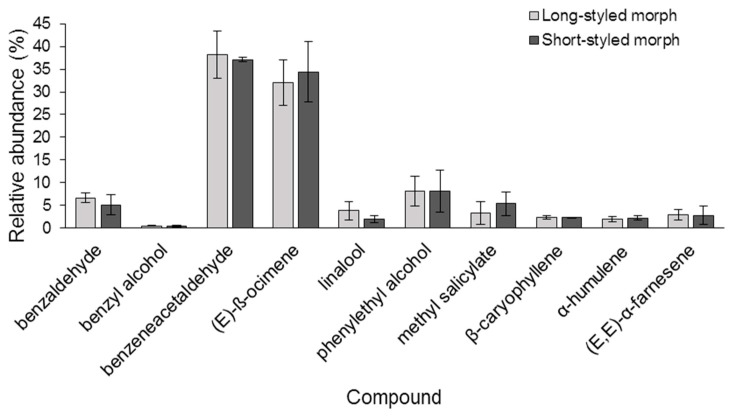
Mean (±SE) of the relative abundance of floral volatile compounds emitted by long- and short-styled morphs of *Guettarda scabra*. N_long-styled samples_ = 2 and N_short-styled samples_ = 2.

Floral scent was found to contain 10 VOCs from two chemical classes: benzenoids and terpenes. These were found in the scent samples of both long- and short-styled floral morphs of *G. scabra* (Table 1, Figure 3). Floral scent samples of the long- and short-styled morphs had identical VOCs and there was no difference in the relative abundance of each compound (Fisher’s exact tests *p* > 0.05 for linalool, β-caryophyllene, (*E*,*E*)-α-farnesene, α-humulene, methyl salicylate, and benzyl alcohol). Benzeneacetaldehyde (also known as phenylacetaldehyde) *X*^2^_2_ = 0.02, *p* = 0.88; (*E*)-β-ocimene *X*^2^_2_ = 0.09, *p* = 0.76; phenylethyl alcohol (also known as 2-phenylethanol) *X*^2^_2_ = 0, *p* = 1; benzaldehyde *X*^2^_2_ = 0.35, *p* = 0.55) (Figure 3 and Figure 4).

The scent of *G. scabra* was strongly dominated by two VOCs, benzeneacetaldehyde and (*E*)-β-ocimene (Figure 3 and Figure 4), with each of them contributing more than 30% of the total chromatogram area (Figure 4). Phenylethyl alcohol accounted for 8% of the chromatogram area, whereas the remaining compounds were present in smaller relative proportions. Among the 10 VOCs emitted by the flowers, benzyl alcohol was the smallest peak present in the chemical profile, representing less than 0.5% of the total area.

## 3. Discussion

### 3.1. Scent Emission within the Flower

The retention of neutral red in the corolla lobes, anthers, and stigma of both long- and short-styled floral morphs of *G. scabra* suggests that these structures are the primary source of the flower’s scent. The presence of scent-emitting regions in flowers is a common trait in the Rubiaceae family, where many species, including *Psychotria homalosperma* [35], *Hillia parasitica* [16], *Faramea cyanea* [36], *Isertia laevis* [37], *Kadua haupuensis* [38], and *Randia itatiaiae* [39] produce fragrant flowers whose scent is thought to mediate pollinator attraction. While flower scent production can involve the entire blossom, it is often concentrated in specific regions or structures [40], with petals generally being the main source of VOCs responsible for the fragrance [40,41,42].

The emission of scent by the corolla lobes in *G. scabra* would be consistent with previous reports of osmophores in this region in other Rubiaceae species, such as *Pagamea duckei* [43], *Psychotria ipecacuanha* [44], and *Chiococca alba* [45]. Furthermore, the pattern of staining of the corolla lobe is a trait shared with another member of the genus, *G. platypoda*, albeit in this species neutral red is additionally retained by the corolla tube [46]. Although non-glandular structures like anthers and stigmata can retain neutral red [47], their potential contribution to *G. scabra*’s fragrance cannot be dismissed, as they are known to emit odor in many plants [2,40].

Floral scent is critical for guiding pollinators to flowers at long and short distances [10]. However, at close range, scent may also convey information about resource quality for some pollinators [48]. In the case of *G. scabra*, the upper surface of the corolla lobes and adjacent structures emit fragrance, which is beneficial as it maximizes pollinator exposure to the scent while they feed from the flower. This is particularly important for *G. scabra* since it is pollinated by hawkmoths [33], whose foraging efforts per flower seem to be heavily influenced by stimulation of the tip of the proboscis with flower volatiles as they reach for nectar inside the corolla [48].

### 3.2. Floral Scent Composition

Although variation in floral scent can occur among groups of plants within a species, such as sexual types, color phenotypes, and floral morphs [14,49,50], our findings show that both long- and short-styled morphs of *G. scabra* have the same composition and proportion of VOCs. This is consistent with observations made in other distylous Rubiaceae, such as *Luculia pinceana* [51] and *Psychotria homalosperma* [35], in which different floral morphs emit the same chemical compounds, albeit some of them with different proportions in *P. homalosperma*. Floral scents with similar chemical profiles across morphs may promote equal rates of visitation by pollinators to either morph, ultimately leading to similar rates of pollination and perhaps promoting outcrossing between them. In this scenario, having similar fragrances could prevent either morph from becoming dominant, resulting in equal abundance of both floral morphs within populations, as observed in *G. scabra* [52].

Distantly related plant species that interact with the same group of pollinators often exhibit evolutionary convergence in their floral traits (pollination syndromes), including the makeup of their flower fragrance [15,53,54]. The chemical composition of the floral scent of *G. scabra*, as revealed by this study, fits the fragrance profile of plants that commonly attract hawkmoths. This finding is consistent with the recent report that this plant is in fact pollinated by *Xylophanes tersa* (Sphingidae), and likely other nocturnal hawkmoths [33]. Floral scents in many hawkmoth-pollinated plants are generally rich in volatile benzenoids and terpenes, and often contain small amounts of nitrogen-containing compounds [15,16,17]. Common constituents of the floral profile of these plants include methyl benzoate, benzyl alcohol, phenylethyl alcohol, and esters such as methyl salicylate among benzenoids and nerolidol, (*E*)-β-ocimene, farnesene, linalool, and β-caryophyllene among terpenes [15,16,17].

The floral bouquet of *G. scabra* is composed entirely of benzenoids and terpenes, with two compounds, benzeneacetaldehyde and (*E*)-β-ocimene, being the most abundant, followed by phenylethyl alcohol in much lower amounts. These compounds are crucial components of the fragrance of many plant species across different families that depend on hawkmoths for pollination. The codominance of terpenes and benzenoids found in *G. scabra* is also reported in many other plants, although the primary compounds involved may vary [15].

Similar to *G. scabra*, (*E*)-β-ocimene is a dominant component in the aroma of *Clarkia cocinna* (Onagraceae) [55], *Brugmansia × candida* (Solanaceae) [56], *Crinum asiaticum* (Amaryllidaceae) [17], and both *Mirabilis jalapa* and *Selinocarpus parvifolius* (Nyctaginaceae) [7,57]. In some cases, such as in *Platanthera chlorantha* (Orchidaceae) [58] and *Capparis spinosa* (Capparaceae), (*E*)-β-ocimene is often codominant with linalool [59], while in *Dianthus monspessulanus* and *D. superbus* (Caryophyllaceae) this compound shares its dominance with β-caryophyllene [60]. On the other hand, in *Trichosanthes kirilowii* (Cucurbitaceae) the dominant compounds in the fragrance are benzeneacetaldehyde (38.9%) and linalool (23%), a benzenoid and a terpene, respectively [17].

Some of the floral volatiles found in *G. scabra* are also common in other Rubiaceae that attract sphingid moths as pollinators. For example, the floral essential oil in *Psychotria eurycarpa* is dominated by linalool and methyl salicylate [61], while in *Randia mutudae* it is largely composed of linalool and benzyl alcohol [62]. On the other hand, phenylethyl alcohol is found in both species but in much lower amounts. Interestingly, while the relative abundance of benzyl alcohol among the floral volatiles in *G. scabra* is negligible (<1%), in its close relative *G. poasana* the same compound dominates the composition of the essential oil (77%), while the remaining compounds (cinnamyl alcohol, 1-indanol, and phenylethyl alcohol) are less abundant [63]. It is very likely that the relative abundance of individual compounds in floral volatiles and essential oils differs due to the nature of the extraction methods used (headspace versus distillation). In fact, some highly VOCs found using the headspace method may be absent from the essential oil samples [64]. However, the presence of benzyl alcohol in the floral essential oil of *R. mutudae* and *G. poasana* indicates that VOCs were obtained and are therefore present in the floral fragrance.

Although the floral fragrance of *G. scabra* may enhance its attractiveness to hawkmoth pollinators, it does not preclude visits from other insects capable of using floral volatiles as olfactory cues to locate floral resources. For example, in south Florida, flowers of *G. scabra* are visited at night by two species of long-horned beetles (Cerambycidae) that feed on their pollen [34]. In this case, flower scent is likely used by beetles foraging for pollen, since some anthophilous cerambycids are attracted to linalool [65] and to phenylethyl alcohol [66], both compounds present in the fragrance of *G. scabra*. Since *G. scabra* flowers remain open and retain a slight fragrance in the morning, several groups of insects, such as butterflies, wasps, and bees, visit them [34].

The flowers of *G. scabra* are frequented by diurnal insects who rely predominantly on visual and olfactory cues to find floral rewards. Some of the most assiduous visitors are *Heliconius charithonia* (Nymphalidae) butterflies [33], which depend strongly on vision to select which flowers to visit from afar but switch to olfaction once they land on the flower, since their feeding behavior is triggered and heavily modulated by flower scent [67]. The relevance of floral volatiles emitted by *G. scabra* as foraging cues for nymphalid butterflies is also seen in *H. melpomene*, which use benzyl alcohol and linalool to locate food sources [67]. Unlike butterflies, bees utilize mostly visual cues when they are close to flowers, but at a distance they are strongly guided by both visual and olfactory cues [68,69]. Many of the species reported visiting flowers of *G. scabra* [34] are indeed attracted to compounds found in their scent. For example, benzyl alcohol, (*E*,*E*)-α-farnesene, and linalool are attractive to *Apis mellifera*, benzeneacetaldehyde to Halictid bees, and methyl salicylate and phenylethyl alcohol attract Euglossini bees [70]. Most notably, *Euglossa dilemma*, an exotic species found on flowers of *G. scabra* [34], visits plants in south Florida collecting fragrant compounds that closely resemble those found in its mutualistic orchids in the neotropics [71]. Some of these compounds include (*E*)-β-ocimene, linalool, β-caryophyllene, humulene, and (*E*,*E*)-α-farnesene, all present in *G. scabra* floral fragrance.

The fragrance profile of *G. scabra* is consistent with the floral-scent bouquets of plants that attract nocturnal moths as pollinators. It is likely that some of the compounds present in *G. scabra* flower scent have a dual function: luring pollinators and deterring antagonists. For example, both linalool and β-caryophyllene are known to be attractive to different groups of insect pollinators [15]. At the same time, these compounds have been found to also serve as a deterrent to flower-feeding insects for *Convolvulus arvensis* (Convolvulaceae) and *Melilotus alba* (Fabaceae) [72]. Similarly, 2-phenylethanol (phenylethyl alcohol) released by *Polemonium viscosum* (Polemoniaceae) at low concentrations serves as an attractant to its pollinator, the bumblebee *Bombus balteatus*, while at high concentrations it repels flower-damaging ants (*Formica neorufibarbis*) [73]. Lastly, *Petunia × hybrida*’s methyl benzoate attracts hawkmoths while simultaneously deterring attacks by flower-feeding insects [74].

## 4. Materials and Methods

### 4.1. Study Species

*Guettarda scabra* (L.) Vent. (Rubiaceae) is an evergreen shrub native to the Caribbean region. It can be found from southern Florida in the United States to northern parts of Colombia and Venezuela [75,76,77]. In south Florida, *G. scabra* typically blooms from May to July and is restricted to the few fragments of pine rockland and hardwood hammock habitats remaining today [24]. Although this species exhibits a special case of distyly in which stigma and anther height vary continuously, it is still possible to recognize two distinct floral morphs (short- and long-styled flowers) within its populations due to the bimodal distribution of stigma–anther separation [52] (Figure 1). The flowers of *G. scabra* are sphingophilous, exhibiting traits usually associated with the attraction of nocturnal moths of the Sphingidae family. These traits include a large, white, tubular corolla with a strong scent, traditionally described as sweet [2,30] (Figure 1). The flowers typically open late in the evening and remain open all night, emitting a strong fragrance that is noticeable from a distance. By the following morning, the scent is detectable only at close range, and the flowers are wilted by noon.

### 4.2. Plant Material

During the late afternoon (between 18:00 and 19:00 h), branches with floral buds ready to open were obtained from long- and short styled-morphs of *G. scabra* growing in two pine rockland fragments in Miami-Dade County, Florida, US: Long Pine Key, located in Everglades National Park (ENP) (25°24′13.2″ N 80°39′33.2″ W), and at Larry and Penny Thompson Memorial Park (LPT) (25°35′55” N 80°23′55″ W). The cut end of each branch was kept in fresh water and immediately transported to the Plant Chemical Ecology Lab at Florida International University to collect floral scent. Material from ENP was collected on 22 June 2019 and from LPT on 21 July 2020.

### 4.3. Localization of Scent-Emitting Regions within the Flower

To assess the location of osmophores (scent-emitting regions) in both short- and long-styled morphs, detached flowers were stained with neutral red [10,78], which is selectively absorbed and retained by undamaged osmophore tissue [40]. Neutral red is a highly effective stain for osmophores due to two key factors. First, osmophores have a highly permeable cell wall, which allows neutral red to enter the cell easily [40]. Second, immediately after flower anthesis, osmophores undergo significant changes in their metabolic activity, becoming highly vacuolated to support scent production and release [79]. Neutral red cations rush into these vacuoles and become locked in them due to the high affinity of vacuoles for positively charged molecules [40]. This results in strong staining of osmophores, making them clearly visible.

Ten plants of each floral morph were selected and two freshly-opened flowers were collected from each individual, for a total of 20 flowers per morph. These flowers were submerged in an aqueous solution of neutral red (0.1% in distilled water) for 20 min. Afterward, flowers were rinsed with distilled water to remove excess dye and then photographed.

### 4.4. Collection and Chemical Analysis of Floral Scents

Scent samples were collected at night (between 20:30 and 21:30 h) from freshly-cut flowers in the lab (23 °C, 60% RH) rather than in the field, where nocturnal temperatures and relative humidity ranged from 25–29 °C and 67–100%. Such controlled conditions reduce the risk of contamination and interference in VOC collection efficiency related to fluctuations in relative humidity. Additionally, preliminary analysis showed no difference in the abundance and composition of VOCs between field collections and laboratory collections.

**Figure 5:**
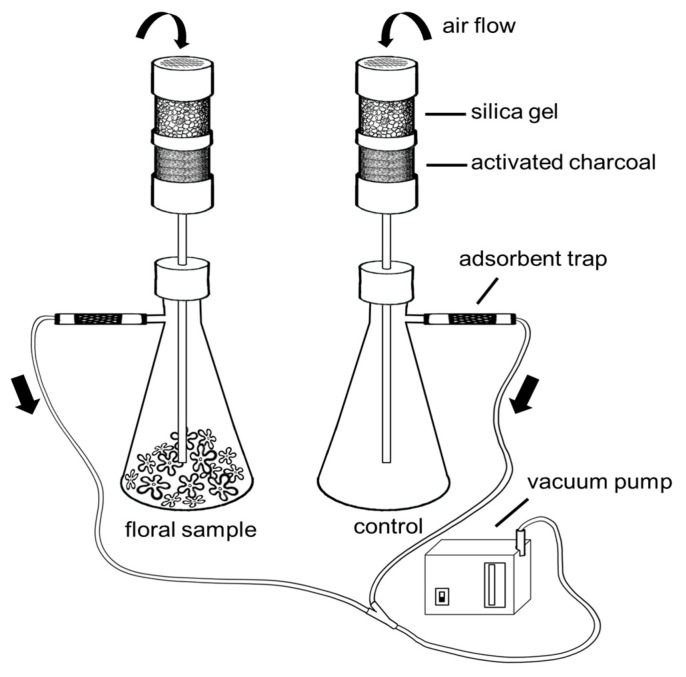
Dynamic headspace setup for the collection of floral scent from detached flowers. Headspace chambers consisted of 500 mL Erlenmeyer flasks into which organic volatile compounds (VOCs) diffused. Using a vacuum pump, air was forced into two flasks, one with the freshly-cut flowers (floral sample) and the other empty (environmental control). Air flowing in was filtered using cartridges containing silica gel and activated charcoal, effectively removing any moisture and contaminants present. The clean air entered the flask and mixed with any VOCs present inside. This enriched air was then forced to flow through adsorbent traps where VOCs were retained, while clean air exited the system through the pump.

Branches from short- and long- styled floral morphs were collected from 52 individuals: 12 plants/morph at LPT and 14 plants/morph at ENP, to account for variation among individuals. Once in the lab, flowers were carefully removed from branches and grouped according to each floral morph (short- and long-styled) and sampling location (LPT and ENP). Samples from LPT contained 80 flowers per morph (6–10 flowers/plant) and those from ENP contained 81 flowers per morph (3–8 flowers/plant). Groups of flowers were placed in 500 mL Erlenmeyer flasks to collect their VOCs using dynamic headspace sampling [10] (Figure 5). Fragrant headspace air in the flask was allowed to reach equilibrium for 15 min, and then air was drawn from the flask using a mini membrane pump (Gilian BDX-II personal air sampling pump, Sensidyne^®^, St. Petersburg, FL, USA) at a flow rate of 500 mL/min for 60 min. Air removed from the flask was passed through a 6 × 70 mm adsorbent glass tube (Zefon International^®^, Ocala, FL, USA), which contained a mixture of 45 mg Tenax TA (divided in two sections of 30/15 mg), and 30 mg of active charcoal (mesh 20/40), where VOCs were trapped. Surrounding air was collected simultaneously as negative control to account for the presence of ambient contaminants (Figure 5). Upon collecting headspace samples, volatiles were eluted from the adsorbent tubes with 1 mL of a solution of hexane and acetone (10:1) and stored at −20 °C until they were analyzed.

Eluted volatile samples were analyzed on a coupled gas chromatography–mass spectrometry system (GC–MS) (7890B/5977A series GC/MSD, Agilent Technologies) equipped with a HP-5 ms capillary column (5% phenyl methyl silox; 30 m, 0.25 mm i.d., 0.25 μm film thickness; Agilent Technologies). For each sample, 1.2 μL was injected into a 4 mm ID single taper inlet liner with wool (Restek) using a split injection technique (split ratio, 1:1). The carrier gas was helium with a head pressure of 9.7 psi and a flow rate of 1.2 mL/min flow; the electron impact ion source (EI) was 70 eV, full scan (50–650 amu). Inlet and MSD temperatures were kept constant at 250 °C and 260 °C, respectively. The GC oven initial temperature was held at 50 °C for 2 min, then increased 5 °C/min until reaching 75 °C, and finally 10 °C/min until reaching 240 °C, where it was held for 2 min.

VOCs were identified by comparing mass-spectral fragmentation patterns with those in the 2017 National Institute of Standards and Technology (NIST) libraries using NIST MS (search program version 2.3). To determine the relative amounts of volatiles in the chromatogram of a sample, the peak area of each compound was calculated as a proportion in relation to the total peak area on that gas chromatogram, allowing comparison among samples. Compounds found in similar abundance in both the control and experimental samples were excluded from the analysis. Floral compounds found were classified based on Knudsen and collaborators [11].

### 4.5. Statistical Analysis

Proportions of VOCs present in the floral bouquets of long- and short-styled morph samples were compared using Pearson’s chi-square test or Fisher’s exact test (if expected cell frequencies were less than 5) (*p* < 0.05). All analyses were performed using R version 4.1.1 [80].

## 5. Conclusions

The fragrance of the flowers of *G. scabra* consists entirely of benzenoid and terpenoid compounds and is characterized by the dominance of benzeneacetaldehyde and (*E*)-β-ocimene. Its chemical profile fits that of night-blooming plants pollinated by nocturnal hawkmoths. The fact that scent emission comes from the corolla lobes of flowers guarantees its exposure to the hawkmoth’s mouthparts during foraging. This finding suggests that flower scent in *G. scabra* could help boost pollinators’ foraging efforts, increasing the chance of successful pollen transfer. Despite having two different floral morphs (short- and long-styled), there is no difference in the chemical profiles of their floral scents, indicating that both floral morphs are likely attracting the same pollinators equally. While the floral scent in *G. scabra* serves the primary role of attracting nocturnal hawkmoth pollinators, it may also be used by other nocturnal and diurnal insects to locate floral resources.

These findings establish baseline knowledge of the floral chemical ecology of *G. scabra*. This study presents an example where floral scent chemistry validates predictions based on flower morphology in a sphingophilous plant. A deeper understanding of the traits involved in the association between plants and their pollinators, as presented here, is necessary to develop strategies toward the conservation of plants and pollinators in endangered habitats such as the pine rockland. Further research in *G. scabra* should address the temporal dynamics of fragrance release, an unknown and yet relevant aspect of the mutualistic relationship between this plant and its hawkmoth pollinators.

## Figures and Tables

**Table 1:** Relative abundances (%) of floral scent constituents of long- and short-styled flower morphs of *Guettarda scabra* (Rubiaceae) identified in dynamic headspace samples. RT = retention time. Flowers were collected at two sites in south Florida: Long Pine Key, Everglades National Park (ENP) and Larry and Penny Thompson Memorial Park (LPT). Samples (A) and (B) were obtained from 14 plants and 81 flowers (3–8 flowers/plant) each. Samples (C) and (D) were obtained from 12 plants and 80 flowers (3–6 flowers/plant) each. * Compounds were classified based on Knudsen and collaborators [11].

Chemical Class		Compound *	RT (min)	Long-styled Morph		Short-styled Morph	
				ENP (A)	LPT (C)	ENP (B)	LPT (D)
Benzenoids	Aldehydes	benzaldehyde	7.26	5.59	7.76	7.35	2.93
		benzeneacetaldehyde	9.25	43.46	32.92	37.64	36.56
	Alcohols	benzyl alcohol	9.09	0.56	0.45	0.18	0.72
		phenylethyl alcohol	10.67	11.48	4.79	3.47	12.70
	Esters	methyl salicylate	12.12	0.82	5.81	2.72	8.04
Terpenes	Monoterpenes	(*E*)-β-ocimene	9.34	27.05	37.04	41.04	27.82
		linalool	10.39	5.78	1.86	2.75	1.26
	Sesquiterpenes	β-caryophyllene	15.50	1.89	2.78	2.40	2.26
		α-humulene	15.94	1.49	2.49	1.71	2.81
		(*E*,*E*)-α-farnesene	16.52	1.88	4.09	0.74	4.90

## Data Availability

The data that support the findings of this study are openly available in the FIU Research Data Portal at https://doi.org/10.34703/gzx1-9v95/2ESGJC.
